# A gene silencing-based approach to tackle fatty liver disease

**DOI:** 10.1016/j.omtm.2024.101198

**Published:** 2024-02-09

**Authors:** Pavel Strnad, Christina Schrader, Nicola Brunetti-Pierri

**Affiliations:** 1 Medical Clinic III, Gastroenterology, Metabolic Diseases and Intensive Care, University Hospital RWTH Aachen, Aachen, Germany; 2 Telethon Institute of Genetics and Medicine (TIGEM), Pozzuoli, Naples, Italy; 3 Department of Translational Medicine, Federico II University, Naples, Italy; 4 Scuola Superiore Meridionale (SSM, School of Advanced Studies), Genomics and Experimental Medicine Program, University of Naples Federico II, Naples, Italy

## Main text

Non-alcoholic fatty liver disease, recently renamed to metabolic dysfunction-associated steatotic liver disease, is a global health problem affecting more than 25% of the population in the Western world. It is driven by metabolic disturbances such as obesity or diabetes leading to fat accumulation in the liver. A subset of individuals with this disorder develop chronic inflammation (i.e. non-alcoholic steatohepatitis [NASH]) and progressive hepatic fibrosis that may ultimately progress into end-stage liver disease and/or hepatocellular carcinoma.^1^ Given its rising prevalence and its major impact on health, NASH became the focus of several pharmaceutical companies, and multiple clinical trials have been initiated.^2^ However, several drug candidates that showed great promise in preclinical studies have failed in the corresponding clinical trials, and to date, there are still no FDA-approved drugs for NASH. While these disappointing results might be related at least in part to the challenge in showing clinical improvements in a slowly progressing disorder, differences between humans and animal models typically used for preclinical studies might be involved as well.^3^ For example, currently used rodent models might not recapitulate the underlying complex etiology of human NASH. Genetic variants responsible for lipid processing in hepatocytes, such as patatin-like phospholipase domain-containing protein 3 (*PNPLA3*), have emerged as important players in NASH progression, but these findings are not yet translated into commonly used experimental NASH models.^1^ While the C/G rs738409 variant in *PNPLA3* constitutes the most established genetic driver of NASH, mice knocked in for the variant^4^ do not fully recapitulate the human disorder. There are at least two possible explanations for the discrepancy between mouse and human NASH. First, there are intrinsic differences in metabolism rate and pathways between mouse and human hepatocytes. Second, NASH is a genetically complex disorder that results from the contribution of a vast array of distinct genetic variants.

**Figure 1:**
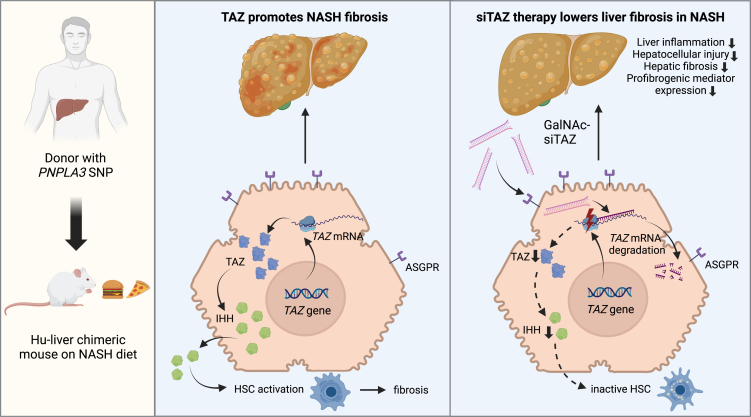
Silencing of the transcriptional regulator TAZ in humanized liver as an approach to tackle fatty liver disease Figure was created with BioRender. PNPLA3, patatin-like phospholipase domain-containing protein 3; SNP, single-nucleotide polymorphism; NASH, non-alcoholic steatohepatitis; Hu, human hepatocytes; TAZ, transcriptional coactivator with PDZ-binding motif; siTAZ, small interfering RNA against TAZ; GalNAc, N-acetylgalactosamine conjugation; ASGPR, asialoglycoprotein receptor; IHH, Indian hedgehog; HSC, hepatic stellate cell.

Mice with humanized livers originating from cells of donors carrying all the genetic makeup resulting in increased susceptibility to NASH is an attractive option to overcome the limitations of currently available mouse models. Such mice are generated using transgenic, immunodeficient mice whose hepatocytes are injured by overexpression of transgenic factors, thereby promoting the outgrowth of transplanted hepatocytes from the donor.^5^ In a recent study published in *Molecular Therapy Methods and Clinical Development*, Wang and colleagues^6^ used a severe combined immunodeficiency mouse strain with chronic liver injury due to transgenic overexpression of urokinase-type plasminogen activator to facilitate engraftment and repopulation by human hepatocytes from a donor carrying the *PNPLA3* risk allele (Figure 1). Despite the strengths, this approach is not without limitations. The mice with humanized liver have a severely impaired immune system, and several cells in the liver (e.g., Kupffer cells and stellate cells) remain of murine origin. Therefore, these models should be used as a complementary, rather than a standalone, tool. This is the case of the current study that extended findings from transgenic animals subjected to a well-established experimental NASH model. However, whether this stepwise process with independent mouse models can be effective for the selection of compounds for clinical trials will require further studies.

The study also raises questions on how broad the results achieved in this mouse model are. Will the results be applicable to other individuals with other risk factors besides *PNPLA3*? Will the results be applicable to other individuals with the same *PNPLA3* risk allele, given that this is likely not the only factor predisposing to NASH? Additional questions are related to the *TAZ* gene that was targeted by Wang and colleagues^6^ (Figure 1). What is the prevalence of *TAZ* upregulation in NASH and, thus, the population of patients that can be treated by drugs inhibiting or downregulating TAZ? Further research is needed to address these important questions.

In the mice with humanized NASH liver, Wang and colleagues^6^ employed a small interfering RNA (siRNA) as a second trendsetting approach. siRNAs are ∼20 base pair long double-stranded RNAs that take advantage of the endogenous virus defense system and are loaded on the so-called RNA-induced silencing complex to mediate long-term suppression of their target sequence.^7^ Unlike antisense oligonucleotides, siRNAs require an active mean for delivery to cells. While this requirement hampers their clinical translation, it greatly increases their specificity and minimizes potential adverse effects. Hepatocytes are the preferred siRNA targets since conjugation with N-acetyl-galactosamine enables their specific entry via asialoglycoprotein receptor (Figure 1). This mechanism is used by nearly all currently approved siRNA drugs that target disorders such as porphyria, primary hyperoxaluria, lipid metabolism, or amyloidosis.^7^ In addition, clinical trials are ongoing to investigate siRNA efficacy in alpha1-antitrypsin deficiency and hepatitis B but also NASH.^7^^,^^8^ While current NASH approaches focus on the established genetic risk alleles,^7^^,^^8^ experimental studies identified attractive siRNA compounds^9^^,^^10^ that deserve further evaluation. Within this context, the current article by Wang and colleagues^6^ shows the importance of advanced experimental models to promote the selection of siRNA compounds for further clinical testing. The study also put forward the ultimate form of precision medicine by which the liver of the patient is reconstituted into a mouse for testing of a drug candidate, which can be given back to the patient once its efficacy has been shown in the experimental model. However, repeating this procedure for any single patient appears not practical given the large number of affected individuals with NASH and the currently available number of laboratories with the needed expertise on humanized mouse models. Alternatively, the choice of the drug can be made upon genetic screen for the *PNPLA3* or combined polygenic risk score that is found to be responsive to TAZ siRNA.

In conclusion, the approach by Wang and colleagues^6^ might be replicated for other leading preclinical drug candidates and has the potential to provide an effective patient-specific armamentarium of therapeutics to combat ongoing NASH epidemics.
